# From bench to bedside: the role of gastrointestinal stem cells in health and disease

**DOI:** 10.1186/s41232-025-00378-1

**Published:** 2025-05-28

**Authors:** Xiaopeng Bai, Eikichi Ihara, Yoshimasa Tanaka, Yosuke Minoda, Masafumi Wada, Yoshitaka Hata, Mitsuru Esaki, Haruei Ogino, Takatoshi Chinen, Yoshihiro Ogawa

**Affiliations:** 1 Department of Medicine and Bioregulatory Science, Graduate School of Medical Sciences, Kyushu University, Fukuoka, Japan; 2 Faculty of Health Sciences Farncombe Family Digestive Health Research Institute, McMaster University, Hamilton, Canada; 3 Department of Gastroenterology and Metabolism, Graduate School of Medical Sciences, Kyushu University, Fukuoka, Japan

**Keywords:** Gastrointestinal stem cells, Tissue regeneration, Cancer stem cells, Therapeutic applications, Signaling pathways

## Abstract

The gastrointestinal (GI) tract constitutes a sophisticated system integral to digestion, nutrient absorption, and overall health, with its functionality predominantly hinging on the distinctive properties of diverse stem cell types. This review systematically investigates the pivotal roles of stem cells across the esophagus, stomach, small intestine, and colon, emphasizing their crucial contributions to tissue homeostasis, repair mechanisms, and regeneration. Each segment of the GI tract is characterized by specialized stem cell populations that exhibit distinct functional attributes, highlighting the necessity for tailored therapeutic approaches in the management of gastrointestinal disorders.

Emerging research has shed light on the functional heterogeneity of GI stem cells, with ISCs in the small intestine displaying remarkable turnover rates and regenerative potential, whereas colonic stem cells (CSCs) are essential for the preservation of the colonic epithelial barrier. The intricate interplay between stem cells and their microenvironment—or niche—is fundamentally important for their functionality, with critical signaling pathways such as Wnt and Notch exerting substantial influence over stem cell behavior. The advent of organoid models derived from GI stem cells offers promising avenues for elucidating disease mechanisms and for the preclinical testing of novel therapeutic interventions.

Despite notable advancements in foundational research on GI stem cells, the translation of these scientific discoveries into clinical practice remains limited. As of 2025, Japan’s clinical GI disease guidelines do not endorse any stem cell-based therapies, underscoring the existing disconnect between research findings and clinical application. This scenario accentuates the urgent need for sustained efforts to bridge this divide and to cultivate innovative strategies that synergize stem cell technology with conventional treatment modalities.

Future investigations should be directed toward unraveling the mechanisms that underpin stem cell dysfunction in various gastrointestinal pathologies, as well as exploring combination therapies that harness the regenerative capacities of stem cells in conjunction with immunomodulatory treatments. By fostering collaborative endeavors between basic researchers and clinical practitioners, we can deepen our understanding of GI stem cells and facilitate the translation of this knowledge into effective therapeutic interventions, ultimately enhancing patient outcomes in gastrointestinal diseases.

## Background

The gastrointestinal (GI) tract is an essential and complex system that plays a critical role in the processes of digestion, nutrient absorption, and waste elimination. It comprises a sequence of interconnected hollow organs, including the esophagus, stomach, small intestine, and colon, each fulfilling specialized functions within the digestive continuum. The efficacy of GI function is augmented by a range of accessory organs, such as the liver, pancreas, and gallbladder, which secrete vital enzymes and substances necessary for digestion and the assimilation of nutrients.

A pivotal aspect of maintaining gastrointestinal health is the presence of various populations of stem cells strategically located throughout the GI tract. These stem cells are integral to tissue homeostasis, facilitating the continuous renewal and repair of the epithelial lining in response to injury and environmental challenges. Emerging research has revealed that during repair, adult GI stem cells transiently adopt a fetal-like transcriptional state—a phenomenon termed *fetal reversion*—which enhances their plasticity and regenerative capacity [1, 2]. This process involves the reactivation of developmental signaling pathways, such as Wnt and YAP/TAZ, and suppression of differentiation programs, enabling tissues to regain embryonic-like repair mechanisms [3, 4]. For instance, collagen type I in the extracellular matrix (ECM) facilitates mechano-transduction by binding to integrin receptors on stem cells, triggering downstream activation of YAP/TAZ transcription factors. These factors orchestrate a transcriptional program that silences adult lineage-specific genes and re-activates fetal markers, thereby restoring developmental plasticity [1, 3].

Distinct regions of the GI tract are characterized by unique stem cell populations, specialized to accommodate the specific physiological requirements of each area. For example, gastric stem cells in the stomach exhibit different properties and functions compared to those found in the small intestine and colon, reflecting their specialized roles in preserving the integrity and functionality of these tissues.

While foundational research on GI stem cells has predominantly utilized animal models—providing significant insights into GI biology—such models may not entirely encapsulate the complexities inherent in human physiology. The expertise of gastroenterologists offers a unique vantage point in this domain, as they possess endoscopic access to the entire GI tract, extending from the esophagus to the rectum. This access facilitates the collection of human tissue samples that can advance research directly pertinent to human health. In particular, studies leveraging organoids derived from human GI stem cells, pioneered by Yui et al. [5], who demonstrated the first successful transplantation of cultured colon organoids into damaged murine colons to regenerate functional epithelium, are emerging as promising methodologies for elucidating the intricacies of GI biology and the pathophysiology of related diseases.

By bridging the divide between animal studies and human-centric research, especially through the utilization of organoid models, we can significantly enhance our understanding of the multifaceted roles that stem cells play within the GI tract. This integrative approach not only contributes to the development of innovative therapeutic strategies but also addresses the pressing need for translational research that is directly relevant to improving human health outcomes. As we explore the interplay between GI stem cells and various gastrointestinal disorders, our aim is to contribute meaningfully to the advancement of clinical practices and to enhance patient care within the field of gastroenterology.

## Esophageal stem cells

### Development and homeostasis

The esophagus develops from the anterior foregut endoderm, sharing a common embryological origin with the respiratory system. Throughout its development, the esophageal epithelium transitions from a simple columnar to a stratified squamous architecture, a transformation accompanied by mesenchymal differentiation into muscle layers. Investigations utilizing animal models and human pluripotent stem cell (hPSC) differentiation have provided insights into the roles of various signaling pathways, including bone morphogenetic protein (BMP) signaling, as well as transcription factors such as SOX2, in esophageal development, respiratory-esophageal separation, and epithelial morphogenesis. These signaling pathways, essential during ontogeny, can also be reactivated under pathological conditions affecting the mature esophagus and its stem cell populations [6, 7].

The epithelium maintains a crucial equilibrium between cellular proliferation and differentiation in the adult esophagus. This is characterized by the activity of basal cells, which proliferate, migrate toward the lumen, differentiate, and ultimately undergo apoptotic cell death. Although the precise identification and characterization of esophageal stem cells remain complex challenges, recent studies have suggested the existence of a potential stem cell population localized within the basal cell compartment. These cells exhibit self-renewal capabilities, as demonstrated through clonogenic assays, three-dimensional organotypic cultures, and in vivo epithelial reconstitution following injury. Importantly, these putative stem cells are capable of giving rise to both undifferentiated and differentiated progeny, underscoring their significant regenerative potential [8, 9].

Recent advancements in research methodologies, including bromodeoxyuridine (BrdU) label-chase experiments, single-cell RNA sequencing (scRNA-seq), and DNA methylation profiling in rat models and organoid systems, have elucidated a subpopulation of slow-cycling or quiescent stem cells within the basal layer of the esophagus. These quiescent cells are characterized by elevated levels of hemidesmosomes (HDs) and diminished Wnt signaling activity. Trajectory analysis from scRNA-seq data suggests that these quiescent basal cells initiate critical cell fate decisions, leading to the generation of proliferating and differentiating cells within the basal layer, which subsequently undergo further differentiation in supra-basal and differentiated layers. Disruptions in HD expression or Wnt signaling can significantly impair this process, thereby affecting organoid development and highlighting the intricate interplay between these factors in maintaining esophageal homeostasis [9]. Furthermore, investigations utilizing mouse models have uncovered a heterogeneous population of basal cells exhibiting varying degrees of stem cell potential, which is regulated by signaling pathways including Sox2, Wnt, and BMP, indicating the presence of a non-quiescent stem cell population within the esophageal basal epithelium [6].

### Esophageal diseases and therapeutic applications

Esophageal stem cells have therapeutic potential in treating various pathologies, building on their established roles in homeostasis and regeneration. Disruptions in these processes can lead to conditions such as post-surgical complications, radiation damage, and esophageal cancer.

The esophageal cancer stem cell (ECSC) hypothesis posits a pivotal role for mutated stem cells in the progression of esophageal cancer and their contribution to therapeutic resistance. ECSCs can be distinguished by specific molecular markers, including aldehyde dehydrogenase 1 (ALDH1) and leucine-rich repeat-containing G protein-coupled receptor 5 (Lgr5), as well as by alterations in key signaling pathways, notably the Wnt/β-catenin and Notch pathways. These characteristics render ECSCs critical targets for therapeutic intervention. Innovative strategies aimed at disrupting these signaling cascades, modulating microRNA expression, and targeting hypoxic microenvironments associated with ECSCs hold considerable promise for enhancing treatment efficacy and outcomes in esophageal cancer [10, 11].

**Fig. 1:**
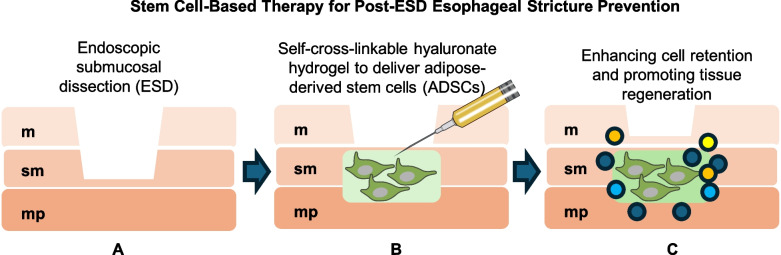
Stem cell-based therapy for post-ESD esophageal stricture prevention. **A** Schematic representation of endoscopic submucosal dissection (ESD) in the esophagus, involving the removal of mucosal (m), submucosal (sm) above muscularis propria (mp) layers. **B** Application of a self-cross-linkable hyaluronate hydrogel loaded with adipose-derived stem cells (ADSCs) to the resection site. The hydrogel enhances ADSC retention and promotes tissue regeneration. (C) Outcome of ADSC-hydrogel therapy, demonstrating reduced stricture formation and restored epithelial integrity across the mucosal, submucosal, and muscularis propria layers. This approach mitigates post-ESD complications by leveraging ADSC-mediated regenerative mechanisms, as demonstrated in preclinical models

Stem cell therapies also offer potential for conditions resulting in strictures or tissue damage. For instance, endoscopic submucosal dissection (ESD) for early neoplasia can lead to post-operative stenosis, where conventional treatments often fall short. Innovative approaches, such as using a self-cross-linkable hyaluronate hydrogel to deliver adipose-derived stem cells (ADSCs), have significantly reduced stricture formation in animal models by enhancing cell retention and promoting tissue regeneration [12, 13] (Fig. 1). Additionally, catechol-functionalized hyaluronic acid hydrogels encapsulating human mesenchymal stem cell spheroids (MSC-SPs) have been tested to counteract radiation-induced esophageal fibrosis. This method provided a supportive microenvironment for MSC-SPs, resulting in improved tissue structure, reduced fibrosis, and enhanced epithelial regeneration [14].

Overall, these studies highlight the potential of stem cell-based therapies, particularly when integrated with advanced delivery systems, in treating various esophageal disorders. Ongoing research focused on targeting ECSCs and optimizing delivery methods holds promise for advancing therapeutic options in this field.

## Gastric epithelial stem cells

### Development and homeostasis

Gastric epithelial stem cells play a pivotal role in the development, maintenance, and regeneration of the stomach lining. These cells predominantly inhabit two distinct anatomical regions of the stomach: the corpus and the pylorus/antrum. Each region is characterized by unique types of stem cells with specific markers and regulatory mechanisms, underscoring their functional diversity [15].

In the pylorus and antrum, Lgr5 + stem cells are located at the base of the glands. These cells engage in symmetric division, facilitating continuous renewal of the epithelial layer, a process regulated by Wnt signaling pathways. They are identified by the expression of multiple markers, including Lgr5, CCKR2, Axin2, and AQP5 [16]. This stem cell population is integral to the regeneration of the gastric epithelium, ensuring a robust response to the cellular turnover dictated by the stomach's harsh acidic environment. Conversely, the corpus presents a distinct profile of gastric stem cells, which are situated near the lumen in the isthmus region. Unlike their counterparts in the pylorus and antrum, corpus stem cells do not rely on Wnt signaling and do not express Lgr5. Instead, they are characterized by markers such as TFF2 mRNA, Mist1 +, and Troy + cells [15, 17]. This divergence highlights the specialized mechanisms adapted by different stem cell populations to fulfill their roles in gastric physiology.

During embryonic development, gastric epithelial stem cells are instrumental in establishing the stomach’s regionalization and patterning. Early gastric progenitors give rise to all differentiated cell lineages present in the adult glandular stomach, thereby laying the groundwork for the organ's complex architecture and functionality. As development transitions to adulthood, these stem cells continue to be critical for homeostasis and tissue regeneration, particularly in response to injury or cellular loss [18]. In the adult stomach, the continuous renewal of the gastric epithelium is crucial, given the organ’s exposure to an acidic environment that limits the lifespan of epithelial cells [15, 16]. Stem cells in both the pylorus/antrum and corpus demonstrate remarkable plasticity, particularly the corpus stem cells, which can increase their proliferation in response to the loss of specific progeny lineages, such as the acid-secreting parietal cells. This adaptability underscores the sophisticated regulatory mechanisms that govern gastric epithelial turnover [15].

The regulation of gastric epithelial stem cells is orchestrated by an array of signaling pathways. Notably, Notch signaling plays a crucial role in modulating the balance between stem cell proliferation and differentiation within the antrum [19]. While Wnt signaling holds significance in antral stem cell function, its influence is markedly diminished in the corpus [20]. Additionally, factors such as BMPs, Shh, and growth factors including EGF and FGF10 contribute to regulating the processes of self-renewal and differentiation [18, 21, 22].

Recent advances in stem cell research have facilitated the development of gastric organoids derived from adult stem cells, as well as those generated through the differentiation of embryonic stem cells or induced pluripotent stem cells [23]. These organoids serve as valuable experimental models for investigating gastric development, the interactions between host cells and *Helicobacter pylori* (*H. pylori*), and mechanisms underlying malignant transformation [16, 24]. A comprehensive understanding of the complex dynamics governing gastric epithelial stem cells is essential, not only for elucidating the processes of stomach development and homeostasis but also for identifying potential therapeutic strategies for gastric diseases. Ongoing research in this domain continues to reveal the sophisticated nature of gastric epithelial stem cells and their integral roles in ensuring stomach function and overall health.

### Gastric diseases and therapeutic applications

Abnormalities in gastric stem cell regulation are increasingly recognized as critical factors in the development of various diseases, notably gastric cancer. One of the central players in this process is the dysregulation of gastric stem cells, particularly the Lgr5 + stem cells, which significantly contributes to the initiation and progression of gastric cancers and related conditions.

Chronic infection with *H. pylori* is a predominant factor in disrupting normal gastric stem cell function and elevating the risk of gastric cancer. The mechanisms through which *H. pylori* promotes carcinogenesis include the induction of gene mutations, most notably affecting the TP53 gene through the upregulation of activation-induced cytidine deaminase (AID) [25]. This infection also induces aberrant DNA methylation in specific promoter regions, including critical tumor suppressor genes. Notably, such epigenetic changes can persist even after the eradication of the bacterium and are correlated with heightened carcinogenic risk [26]. Furthermore, *H. pylori* infection has been shown to enhance the expression of the stem cell marker Lgr5 in gastric organoid cells, potentially expanding the stem cell pool and paving the way for tumorigenesis [27, 28].

Another vital pathway implicated in gastric cancer development is the Notch signaling pathway, which plays an integral role in regulating gastric stem cell dynamics. Dysregulation of Notch signaling can significantly alter stem cell behavior. For instance, Notch activation has been observed to promote the proliferation of gastric stem and progenitor cells, while its inhibition results in suppressed proliferation. Additionally, the balance of differentiation is affected by Notch dysregulation; when Notch signaling is inhibited, there is an increase in mucous and endocrine cell differentiation, whereas persistent activation tends to reduce differentiation overall. Moreover, continuous Notch activation in Lgr5 + stem cells can lead to gland fission and tissue expansion, potentially contributing to tumor formation and the development of undifferentiated, hyper-proliferative polyps in the gastric antrum [19, 29].

The mTOR signaling pathway has also emerged as a significant player in the landscape of gastric cancer development. Research indicates that Notch activation is associated with increased mTOR signaling; therefore, inhibiting mTORC1 can normalize the heightened proliferation and gland fission driven by Notch. This knowledge reveals therapeutic potential, suggesting that targeting the mTOR pathway could be a promising strategy for addressing Notch-induced gastric abnormalities [30].

Understanding these molecular mechanisms is crucial for the effective development of targeted therapies aimed at gastric cancer stem cells (GCSCs). Identifying specific surface and intracellular markers for GCSCs can aid in creating novel therapeutic approaches. Moreover, the tumor microenvironment surrounding GCSCs contributes to their maintenance and tumor progression through the availability of growth factors that support angiogenesis. Importantly, GCSCs exhibit a heightened capacity for drug resistance due to increased activity of drug efflux pumps and the synthesis of anti-apoptotic factors, making them less susceptible to conventional cancer treatments [31–34].

Gastric stem cell abnormalities, particularly those driven by *H. pylori* infection and the dysregulation of signaling pathways such as Notch and mTOR, play substantial roles in the development of gastric cancer and related diseases. A deeper understanding of these mechanisms is essential for the advancement of effective preventive and therapeutic strategies aimed at combating gastric diseases. As research continues to uncover the complex interplay of these factors, new opportunities for intervention and treatment may arise, offering hope for improved outcomes in gastric cancer management.

## Intestinal stem cells

### Development and homeostasis

Two primary types of intestinal stem cells (ISCs) have been identified: the Lgr5 + crypt base columnar (CBC) cells and the + 4 cells. Lgr5 + CBCs are rapidly proliferating stem cells situated at the base of the intestinal crypts, interspersed among Paneth cells. These cells are distinguished by the expression of the Lgr5 marker and play a crucial role in the majority of epithelial turnover during homeostasis. Lgr5 + ISCs are multipotent and capable of generating all types of intestinal epithelial cells, including absorptive enterocytes, secretory cells (goblet, enteroendocrine, and tuft cells), and Paneth cells [35–37]. The + 4 cells, named based on their position relative to the crypt base, constitute a more quiescent population of stem cells. Often referred to as reserve stem cells, these cells are characterized by the expression of markers such as Bmi1. While Lgr5 + CBCs are primarily responsible for daily epithelial maintenance, + 4 cells become activated in response to injury or stress to the epithelium, highlighting the plasticity of the intestinal stem cell compartment [38].

Intestinal stem cells (ISCs) are fundamental to the continuous renewal of the intestinal epithelium, a process that transpires approximately every 3 to 5 days in mammals. This rapid cellular turnover is critical for preserving the integrity of the intestinal barrier and facilitating optimal nutrient absorption. Lgr5 + crypt base columnar (CBC) cells undergo asymmetric division, resulting in the generation of both stem cells and transit-amplifying (TA) cells. Subsequently, these TA cells differentiate into the diverse specialized cell types that constitute the intestinal epithelium as they migrate upward along the crypt-villus axis. The differentiation process of ISCs is intricately regulated by a myriad of signaling pathways, prominently including the Wnt, Notch, and BMP pathways. These molecular signaling avenues maintain the delicate equilibrium between stem cell self-renewal and differentiation, ultimately ensuring a proper cellular composition within the intestinal epithelium [37, 39, 40].

The intestinal stem cell niche is pivotal for modulating ISC functionality and maintenance. Paneth cells, which represent specialized secretory cells localized at the base of the crypts, are integral components of this niche. These cells secrete crucial factors, such as epidermal growth factor (EGF), transforming growth factor-alpha (TGF-α), Wnt3, and the Notch ligand Dll4, which collectively support ISC functionality [41–43]. The close physical association between Lgr5 + CBCs and Paneth cells is essential for sustaining stem cell activity. In vitro investigations have demonstrated that co-culturing ISCs with Paneth cells substantially enhances the formation of intestinal organoids, underscoring the significance of this cellular interaction. Furthermore, the stem cell niche encompasses various stromal cell populations that provide additional support and contribute regulatory signals to ISCs, thereby accentuating the complexity of this microenvironment [44, 45].

### Intestinal diseases and therapeutic applications

Dysfunction of ISCs is implicated in several gastrointestinal pathologies. For instance, mitochondrial impairments within ISCs may precipitate their transition into dysfunctional Paneth cells, which serves as a predictive marker for the recurrence of Crohn's disease [46]. Furthermore, inflammatory processes can induce metabolic alterations in ISCs, resulting in diminished capacities for differentiation and regeneration, as observed in conditions such as graft-versus-host disease (GVHD) and inflammatory bowel diseases (IBD) [47, 48]. Inflammatory bowel diseases, encompassing Crohn's disease and ulcerative colitis, represent chronic inflammatory conditions of the gastrointestinal tract. These maladies are characterized by recurrent inflammation, which may culminate in complications such as intestinal fistulae, obstruction, and hemorrhage. The continual renewal and repair of the intestinal mucosal epithelium under such circumstances is heavily reliant on the proper functioning of ISCs [49, 50].

**Fig. 2:**
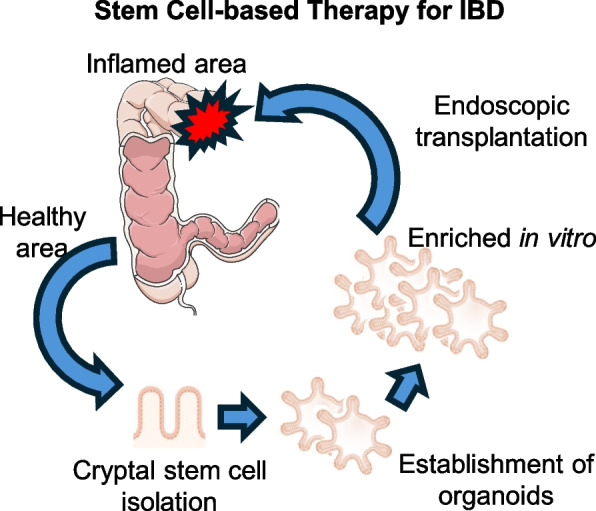
Stem cell-based therapy for IBD. Intestinal crypts and cryptal stem cells can be harvested endoscopically from healthy intestinal mucosa in patients with inflammatory bowel disease (IBD). These cells can then be expanded in vitro using established organoid culture methods. Once they reach the desired number of cells, they can be transplanted to the target site using an endoscopic delivery method

Present therapeutic modalities for IBD predominantly emphasize the suppression of inflammation to avert further tissue damage. Nevertheless, achieving sustained remission and mucosal healing remains a formidable challenge. Emerging stem cell therapies, involving the application of hematopoietic stem cells (HSCs) and mesenchymal stem cells (MSCs), have demonstrated potential in modulating immune responses and facilitating tissue repair. Seminal work by Yui et al. [5] laid the foundation for organoid-based therapies, demonstrating that cultured Lgr5 + colonic stem cells could regenerate functional epithelium in damaged murine colons. Transplanted organoids derived from a single Lgr5 + stem cell integrated seamlessly into injured tissue, forming histologically normal crypts with long-term engraftment (> 6 months). Additionally, the transplantation of ISCs into inflamed mucosal tissue, now extended to human trials using patient-derived organoids, is being investigated as a novel therapeutic strategy aimed at reconstructing the epithelial barrier in IBD [49, 51, 52](Fig. 2). Prospective therapeutic approaches may involve the integration of biological agents with ISC transplantation to augment treatment efficacy for patients with IBD. The development of ISC organoids represents a promising avenue for both research and therapeutic intervention, offering a potential source for the regeneration of damaged intestinal tissues [53]. Moreover, targeting mitochondrial functionality within ISCs emerges as a promising direction for the treatment of intestinal disorders, as the remodeling of mitochondrial activity could enhance ISC performance and overall intestinal health [54]. The application of vitamin D to stimulate ISC differentiation and function is also under investigation as a potential strategy for promoting intestinal epithelial restoration [55].

ISCs play a critical role in sustaining intestinal health, and their dysfunction is associated with a variety of gastrointestinal diseases. While current treatments primarily focus on the suppression of inflammation, future strategies may leverage advancements in stem cell technology and mitochondrial targeting to improve clinical outcomes for individuals afflicted with IBD.

## Colonic stem cells

### Development and homeostasis

Colonic stem cells (CSCs) are essential for preserving the integrity of the colonic epithelial lining, and recent advancements in research have markedly enhanced our comprehension of their characteristics, functions, and implications in disease pathology. This section aims to elucidate these recent findings by comparing CSCs with small intestinal stem cells (ISCs), elucidating their roles in the maintenance of colon epithelium, and investigating their associations with various diseases, particularly inflammatory bowel disease (IBD).

Both CSCs and ISCs serve the fundamental purpose of maintaining and regenerating their respective epithelial tissues; however, they exhibit critical differences. A notable distinction pertains to their differentiation potential: ISCs possess the capability to differentiate into five major cell types, including absorptive enterocytes, goblet cells, enteroendocrine cells, Paneth cells, and tuft cells, whereas CSCs are restricted to three distinct lineages, including colonocytes, goblet cells, and enteroendocrine cells in the colon [49]. Furthermore, a significant disparity exists in their radioresistance; specifically, CSCs demonstrate substantially higher radioresistance in vivo compared to ISCs. This phenomenon can be attributed to variations in cell cycle reentry and the differential susceptibility to mitotic cell death [56]. Additionally, molecular profiling has revealed distinct gene expression patterns between stem cells in the small intestine and those in the colon, with this molecular heterogeneity contributing to the functional differences observed across these regions of the gastrointestinal tract [57].

CSCs are pivotal in sustaining the health and functionality of the colonic epithelium, operating through several key mechanisms. Wnt signaling emerges as a critical regulator of intestinal stem cell activation, with epithelial WNT ligands serving an indispensable role in this process. Furthermore, GLI1-expressing mesenchymal cells constitute a vital Wnt-secreting niche for colon stem cells, thereby underscoring the significance of the stem cell microenvironment. The extracellular matrix (ECM) also contributes significantly, as its mechanical properties influence the fate of intestinal stem cells. The interaction between ECM mechanics and stem cell fate proves crucial for the maintenance of the stem cell niche and the overall functionality of tissue [58, 59]. Lastly, the transcription factor SATB2 has been identified as essential for preserving the identity of colon stem cells and mediating the process of ileum-colon conversion through enhancer remodeling, which emphasizes the importance of epigenetic regulation in safeguarding CSC identity [60].

#### Colonic diseases and therapeutic applications

Abnormalities within CSC populations have been implicated in various pathologies, with particular emphasis on IBD. Investigations utilizing mouse models of IBD-like colitis have demonstrated the persistence of Lgr5 + stem cells, albeit with variable spatial patterns contingent upon the specific model employed. This persistence stands in contrast to the total depletion of these cells observed in the DSS model, indicating that distinct forms of intestinal inflammation may differentially impact CSC populations [61]. Moreover, Krt19 +/Lgr5 − cells have been characterized as radioresistant cancer-initiating stem cells within both the colonic and intestinal contexts, yielding critical insights into the origins of colorectal cancer and facilitating the development of targeted therapeutic strategies [38]. Recent research has highlighted changes in the diversity of colonic epithelial cells in patients with inflammatory bowel disease (IBD). Specifically, there is a positional remodeling of goblet cells, which coincides with the downregulation of WFDC2, an antiprotease molecule expressed by goblet cells that inhibits bacterial growth. These modifications also involve changes in stem cell populations, contributing to disease pathogenesis and potentially revealing new avenues for therapeutic intervention [62]. Additionally, the gut microbiota has emerged as a pivotal factor influencing the intestinal stem cell niche under both homeostatic and inflammatory conditions. A specialized group of microbes, crypt-specific core microbiota, reside in close proximity to ISCs, form a unique microbial niche that directly impacts stem cell function. An enhanced understanding of this intricate relationship may pave the way for innovative approaches aimed at preserving stem cell functionality and treating intestinal disorders [63].

Recent research has considerably advanced our understanding of colonic stem cells, elucidating their unique properties and their significance in both health and disease. These findings offer valuable insights that could inform the development of novel therapeutic strategies targeting CSCs in various gastrointestinal disorders, particularly in the context of IBD.

## Conclusions

**Fig. 3:**
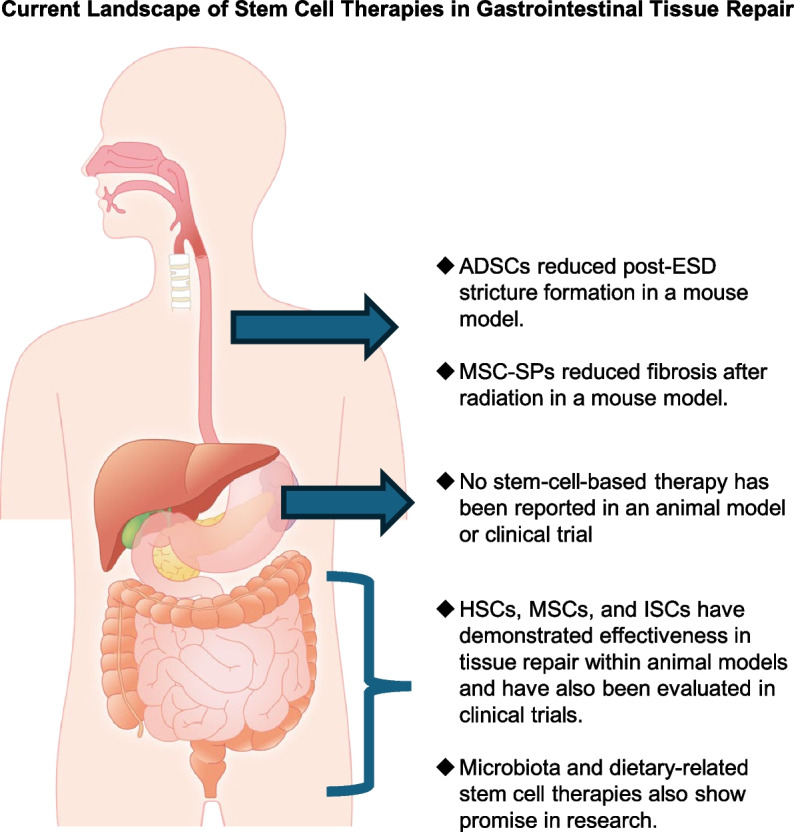
Current landscape of stem cell therapies for gastrointestinal tissue repair. This figure presents an overview of the emerging applications and effectiveness of stem cell therapies throughout various regions of the gastrointestinal tract. Esophagus: adipose-derived stem cells (ADSCs) have demonstrated efficacy in mitigating post-endoscopic submucosal dissection (ESD) stricture formation in mouse models. Mesenchymal stem cell spheroids (MSC-SPs) have been effective in diminishing fibrosis resulting from radiation exposure in animal studies. Stomach: currently, there are no available stem cell-based therapies reported in either animal models or clinical trials, indicating a significant gap in research opportunities. Small intestine and colon: hematopoietic stem cells (HSCs), mesenchymal stem cells (MSCs), and intestinal stem cells (ISCs) have demonstrated their potential for effective tissue repair in animal studies and are under active evaluation in clinical trials. Research into the roles of microbiota and dietary-related stem cell therapies is emerging as a promising avenue for gastrointestinal tissue repair. This figure highlights significant advancements in stem cell research aimed at gastrointestinal tissue repair, while also identifying critical areas that warrant further investigation. The potential applications of various stem cell types and their associated therapies offer promising solutions for addressing gastrointestinal damage and diseases throughout the digestive tract

The gastrointestinal (GI) tract is a highly intricate system whose integrity and function are sustained by the diverse properties of various stem cell populations. This review underscores the specialized roles that distinct regional stem cells—specifically those in the esophagus, stomach, small intestine, and colon—play in tissue homeostasis, repair, and regeneration. A nuanced understanding of these differences is paramount for both basic researchers and clinical practitioners, as it lays the groundwork for the development of targeted therapeutic approaches for gastrointestinal disorders (Fig. 3).

Recent research has illuminated the functional heterogeneity of stem cells within the GI tract. For instance, intestinal stem cells (ISCs) in the small intestine are distinguished by their rapid turnover rates and robust capacity for epithelial regeneration, whereas colonic stem cells (CSCs) exhibit unique characteristics that are essential for maintaining the integrity of the colonic epithelial lining. This functional specialization emphasizes the necessity for tailored therapeutic interventions that correspond with the specific stem cell types implicated in various GI conditions. Notably, emergent stem cell therapies aimed at ISCs for inflammatory bowel disease (IBD) may prove ineffective for pathologies predominantly affecting the colon, where CSCs are more influential.

Furthermore, the interaction between stem cells and their specific microenvironments, or niches, is crucial for their optimal functionality. Advancements in elucidating the signaling pathways, particularly the Wnt and Notch pathways, that govern stem cell dynamics offer significant insights into potential manipulation for therapeutic ends. Targeting these signaling pathways may enhance stem cell functionality, thereby improving patient outcomes in those afflicted with GI disorders. Additionally, the emergence of organoid models derived from GI stem cells represents a promising avenue for both experimental and therapeutic exploration. These models facilitate the investigation of disease mechanisms and the assessment of novel treatments in a controlled setting.

While considerable foundational research on GI stem cells has been conducted using animal models, the prospects for human-based studies are increasingly encouraging. Gastroenterologists have endoscopic access to the entire GI tract, from the esophagus to the rectum, which affords the opportunity to collect human tissue samples for research purposes. Such access enables the development of human-derived organoid studies, which hold the potential to yield insights that are more directly translatable to human health than those derived from animal models.

Despite the advancements in our understanding of GI stem cells, several challenges persist. The translation of basic research findings into clinical applications remains limited, as evidenced by the absence of any recommended stem cell-based therapies in clinical GI disease guidelines in Japan as of 2025. This situation should not be regarded as a setback; rather, it underscores the pressing need for sustained efforts to bridge the gap between research and clinical application. The quest for achieving sustained remission and mucosal healing in conditions such as IBD remains a formidable challenge, highlighting the need for innovative strategies that integrate stem cell technologies with existing therapeutic modalities.

Future research endeavors should prioritize elucidating the mechanisms underlying stem cell dysfunction across a spectrum of gastrointestinal diseases. Additionally, exploring combination therapies that leverage the regenerative capabilities of stem cells in conjunction with immunomodulatory treatments holds significant promise. By fostering collaborative efforts between basic researchers and clinical practitioners, especially through the utilization of human samples and organoid models, we can enhance our understanding of GI stem cells and translate this knowledge into effective therapeutic interventions for patients with gastrointestinal disorders.

In conclusion, the expanding body of literature surrounding GI stem cells highlights their fundamental role in preserving gastrointestinal health and their potential as therapeutic targets. Continued exploration of the distinct properties of various stem cell types, along with their interactions within the GI tract, will be essential for advancing the field and improving patient outcomes.

## Data Availability

Not applicable.

## Abbreviations

ADSC: Adipose-derived stem cells
AID: Activation-induced cytidine deaminase
ALDH1: Aldehyde dehydrogenase 1
Bmi1: B lymphoma Mo-MLV insertion region 1
BMP: Bone morphogenetic protein
BrdU: Bromodeoxyuridine
CBC: Crypt base columnar
CSCs: Colonic stem cells
DSS: Dextran sulfate sodium
ECM: Extracellular matrix
EGF: Epidermal growth factor
ECSC: Esophageal cancer stem cell
GCSCS: Gastric cancer stem cells
GI: Gastrointestinal
GLI1: GLI family zinc finger 1
GVHD: Graft-versus-host disease
HDs: Histone deacetylases
hPSC: Pluripotent stem cell
HSCs: Hematopoietic stem cells
IBD: Inflammatory bowel disease
ISCs: Intestinal stem cells
Krt19: Keratin 19
Lgr5: Leucine-rich repeat-containing G protein-coupled receptor 5
MSC: Mesenchymal stem cells
MSC-SPs: Mesenchymal stem cell spheroids
mTOR: Mechanistic target of rapamycin
SATB2: Special AT-rich sequence-binding protein 2
SOX2: SRY-box transcription factor 2
Wnt: Wingless/integrated

## References

[1] Yui S, et al. YAP/TAZ-dependent reprogramming of colonic epithelium links ECM remodeling to tissue regeneration. Cell Stem Cell. 2018;22(1):35-49 e7. 29249464 10.1016/j.stem.2017.11.001 PMC5766831

[2] Ayyaz A, et al. Single-cell transcriptomes of the regenerating intestine reveal a revival stem cell. Nature. 2019;569(7754):121–5. 31019301 10.1038/s41586-019-1154-y

[3] Kobayashi S, et al. Collagen type I-mediated mechanotransduction controls epithelial cell fate conversion during intestinal inflammation. Inflamm Regen. 2022;42(1):49. 36443773 10.1186/s41232-022-00237-3 PMC9703763

[4] Viragova S, Li D, Klein OD. Activation of fetal-like molecular programs during regeneration in the intestine and beyond. Cell Stem Cell. 2024;31(7):949–60. 38971147 10.1016/j.stem.2024.05.009 PMC11235077

[5] Yui S, et al. Functional engraftment of colon epithelium expanded in vitro from a single adult Lgr5(+) stem cell. Nat Med. 2012;18(4):618–23. 22406745 10.1038/nm.2695

[6] Zhang Y, et al. Development and stem cells of the esophagus. Semin Cell Dev Biol. 2017;66:25–35. 28007661 10.1016/j.semcdb.2016.12.008 PMC5474349

[7] Zhang Y, et al. The development and stem cells of the esophagus. Development. 2021;148(6). 10.1242/dev.193839 PMC8034879 33782045

[8] Kalabis J, et al. A subpopulation of mouse esophageal basal cells has properties of stem cells with the capacity for self-renewal and lineage specification. J Clin Invest. 2008;118(12):3860–9. 19033657 10.1172/JCI35012 PMC2579884

[9] Yang Y, et al. Identification and characterization of stem cells in mammalian esophageal stratified squamous epithelia. J Mol Cell Biol. 2022;14(6). 10.1093/jmcb/mjac038 PMC9669669 35709398

[10] Das PK, et al. Therapeutic Strategies Against Cancer Stem Cells in Esophageal Carcinomas. Front Oncol. 2020;10:598957. 33665161 10.3389/fonc.2020.598957 PMC7921694

[11] Zhou C, et al. Linking cancer stem cell plasticity to therapeutic resistance-mechanism and novel therapeutic strategies in esophageal cancer. Cells. 2020;9(6). 10.3390/cells9061481 PMC7349233 32560537

[12] Chung H, et al. Endoscopically injectable and self-crosslinkable hydrogel-mediated stem cell transplantation for alleviating esophageal stricture after endoscopic submucosal dissection. Bioeng Transl Med. 2023;8(3):e10521. 37206239 10.1002/btm2.10521 PMC10189443

[13] Ye S, et al. Strategies for preventing esophageal stenosis after endoscopic submucosal dissection and progress in stem cell-based therapies. Tissue Eng Part B Rev. 2024;30(5):522–9. 38243787 10.1089/ten.TEB.2023.0316

[14] Kim IG, et al. Regeneration of irradiation-damaged esophagus by local delivery of mesenchymal stem-cell spheroids encapsulated in a hyaluronic-acid-based hydrogel. Biomater Sci. 2021;9(6):2197–208. 33506817 10.1039/d0bm01655a

[15] Mills JC, Shivdasani RA. Gastric epithelial stem cells. Gastroenterology. 2011;140(2):412–24. 21144849 10.1053/j.gastro.2010.12.001 PMC3708552

[16] Xiao S, Zhou L. Gastric stem cells: physiological and pathological perspectives. Front Cell Dev Biol. 2020;8:571536. 33043003 10.3389/fcell.2020.571536 PMC7527738

[17] Wizenty J, Tacke F, Sigal M. Responses of gastric epithelial stem cells and their niche to Helicobacter pylori infection. Ann Transl Med. 2020;8(8):568. 32775369 10.21037/atm.2020.02.178 PMC7347775

[18] Alvina FB, et al. Gastric epithelial stem cells in development, homeostasis and regeneration. Development. 2023;150(18). 10.1242/dev.201494 37746871

[19] Demitrack ES, et al. Notch signaling regulates gastric antral LGR5 stem cell function. EMBO J. 2015;34(20):2522–36. 26271103 10.15252/embj.201490583 PMC4609184

[20] Flanagan DJ, et al. Wnt Signalling in Gastrointestinal Epithelial Stem Cells. Genes (Basel). 2018;9(4). 10.3390/genes9040178 PMC5924520 29570681

[21] Wolffling S, et al. EGF and BMPs govern differentiation and patterning in human gastric glands. Gastroenterology. 2021;161(2):623-636 e16. 33957136 10.1053/j.gastro.2021.04.062

[22] Nyeng P, et al. FGF10 signaling controls stomach morphogenesis. Dev Biol. 2007;303(1):295–310. 17196193 10.1016/j.ydbio.2006.11.017 PMC1864952

[23] Huebner AJ, et al. Dissection of gastric homeostasis in vivo facilitates permanent capture of isthmus-like stem cells in vitro. Nat Cell Biol. 2023;25(3):390–403. 36717627 10.1038/s41556-022-01079-4 PMC13528691

[24] Schlaermann P, et al. A novel human gastric primary cell culture system for modelling Helicobacter pylori infection in vitro. Gut. 2016;65(2):202–13. 25539675 10.1136/gutjnl-2014-307949 PMC4752654

[25] Shimizu T, et al. Accumulation of somatic mutations in TP53 in gastric epithelium with Helicobacter pylori infection. Gastroenterology. 2014;147(2):407-17 e3. 24786892 10.1053/j.gastro.2014.04.036

[26] Muhammad JS, Eladl MA, Khoder G. Helicobacter pylori-induced DNA methylation as an epigenetic modulator of gastric cancer: recent outcomes and future direction. Pathogens. 2019;8(1). 10.3390/pathogens8010023 PMC6471032 30781778

[27] Sigal M, et al. Helicobacter pylori Activates and Expands Lgr5(+) stem cells through direct colonization of the gastric glands. Gastroenterology. 2015;148(7):1392-404 e21. 25725293 10.1053/j.gastro.2015.02.049

[28] He J, et al. Inactivation of the tumor suppressor gene Apc synergizes with H. pylori to induce DNA damage in murine gastric stem and progenitor cells. Sci Adv. 2023;9(46):eadh0322. 37967175 10.1126/sciadv.adh0322 PMC10651120

[29] Demitrack ES, Samuelson LC. Notch regulation of gastrointestinal stem cells. J Physiol. 2016;594(17):4791–803. 26848053 10.1113/JP271667 PMC5009795

[30] Hibdon ES, et al. Notch and mTOR signaling pathways promote human gastric cancer cell proliferation. Neoplasia. 2019;21(7):702–12. 31129492 10.1016/j.neo.2019.05.002 PMC6536707

[31] Bekaii-Saab T, El-Rayes B. Identifying and targeting cancer stem cells in the treatment of gastric cancer. Cancer. 2017;123(8):1303–12. 28117883 10.1002/cncr.30538 PMC5412889

[32] Stojnev S, et al. Gastric cancer stem cells: therapeutic targets. Gastric Cancer. 2014;17(1):13–25. 23563919 10.1007/s10120-013-0254-x

[33] Norollahi SE, et al. Therapeutic approach of Cancer stem cells (CSCs) in gastric adenocarcinoma; DNA methyltransferases enzymes in cancer targeted therapy. Biomed Pharmacother. 2019;115:108958. 31075731 10.1016/j.biopha.2019.108958

[34] Hsieh HL, et al. Molecular mechanism of therapeutic approaches for human gastric cancer stem cells. World J Stem Cells. 2022;14(1):76–91. 35126829 10.4252/wjsc.v14.i1.76 PMC8788185

[35] Kurokawa K, Hayakawa Y, Koike K. Plasticity of Intestinal Epithelium: Stem Cell Niches and Regulatory Signals. Int J Mol Sci. 2020;22(1). 10.3390/ijms22010357 PMC7795504 33396437

[36] Sato T, et al. Paneth cells constitute the niche for Lgr5 stem cells in intestinal crypts. Nature. 2011;469(7330):415–8. 21113151 10.1038/nature09637 PMC3547360

[37] Metcalfe C, et al. Lgr5+ stem cells are indispensable for radiation-induced intestinal regeneration. Cell Stem Cell. 2014;14(2):149–59. 24332836 10.1016/j.stem.2013.11.008

[38] Asfaha S, et al. Krt19(+)/Lgr5(-) cells are radioresistant cancer-initiating stem cells in the colon and intestine. Cell Stem Cell. 2015;16(6):627–38. 26046762 10.1016/j.stem.2015.04.013 PMC4457942

[39] Barker N, et al. Very long-term self-renewal of small intestine, colon, and hair follicles from cycling Lgr5+ve stem cells. Cold Spring Harb Symp Quant Biol. 2008;73:351–6. 19478326 10.1101/sqb.2008.72.003

[40] van der Flier LG, Clevers H. Stem cells, self-renewal, and differentiation in the intestinal epithelium. Annu Rev Physiol. 2009;71:241–60. 18808327 10.1146/annurev.physiol.010908.163145

[41] Xian L, et al. HMGA1 amplifies Wnt signalling and expands the intestinal stem cell compartment and Paneth cell niche. Nat Commun. 2017;8:15008. 28452345 10.1038/ncomms15008 PMC5414379

[42] Quintero M, et al. Intestinal stem cells remodel in response to acute notch inhibition and paneth cell loss. Physiology. 2024;39.

[43] Hou Q, et al. The Research Progress on Intestinal Stem Cells and Its Relationship with Intestinal Microbiota. Front Immunol. 2017;8:599. 28588586 10.3389/fimmu.2017.00599 PMC5440531

[44] Santos AJM, et al. The Intestinal Stem Cell Niche: Homeostasis and Adaptations. Trends Cell Biol. 2018;28(12):1062–78. 30195922 10.1016/j.tcb.2018.08.001 PMC6338454

[45] Choi J, Augenlicht LH. Intestinal stem cells: guardians of homeostasis in health and aging amid environmental challenges. Exp Mol Med. 2024;56(3):495–500. 38424189 10.1038/s12276-024-01179-1 PMC10985084

[46] Khaloian S, et al. Mitochondrial impairment drives intestinal stem cell transition into dysfunctional Paneth cells predicting Crohn’s disease recurrence. Gut. 2020;69(11):1939–51. 32111634 10.1136/gutjnl-2019-319514 PMC7569388

[47] Zhao DC, et al. Inflammatory memory restrains intestinal stem cell (ISC) regeneration after allogeneic stem cell transplantation (SCT). Blood. 2023;142.

[48] Reddy P, et al. Inflammatory memory restrains intestinal stem cell regeneration. Res Sq. 2023:rs.3.rs-2566520. 10.21203/rs.3.rs-2566520/v1.

[49] Hou Q, et al. Intestinal stem cells and immune cell relationships: potential therapeutic targets for inflammatory bowel diseases. Front Immunol. 2020;11:623691. 33584726 10.3389/fimmu.2020.623691 PMC7874163

[50] Tian CM, et al. Stem cell therapy in inflammatory bowel disease: a review of achievements and challenges. J Inflamm Res. 2023;16:2089–119. 37215379 10.2147/JIR.S400447 PMC10199681

[51] Shimizu H, et al. Stem cell-based therapy for inflammatory bowel disease. Intest Res. 2019;17(3):311–6. 31352774 10.5217/ir.2019.00043 PMC6667367

[52] Che Z, et al. Mesenchymal stem/stromal cells in the pathogenesis and regenerative therapy of inflammatory bowel diseases. Front Immunol. 2022;13:952071. 35990688 10.3389/fimmu.2022.952071 PMC9386516

[53] Boye TL, et al. Molecular manipulations and intestinal stem cell-derived organoids in inflammatory bowel disease. Stem Cells. 2022;40(5):447–57. 35365825 10.1093/stmcls/sxac014

[54] Lei X, et al. Regulation of mitochondrial quality control of intestinal stem cells in homeostasis and diseases. Antioxid Redox Signal. 2025;42(10-12):494-511. 10.1089/ars.2023.0489. Epub 2024 Sep 24. 10.1089/ars.2023.0489 39225500

[55] Shaikh NA, et al. 1,25-dihydroxyvitamin D enhances the regenerative function of Lgr5(+) intestinal stem cells in vitro and in vivo. Cells. 2024;13(17). 10.3390/cells13171465 PMC11394149 39273035

[56] Hua G, et al. Distinct levels of radioresistance in Lgr5(+) colonic epithelial stem cells versus Lgr5(+) small intestinal stem cells. Cancer Res. 2017;77(8):2124–33. 28202528 10.1158/0008-5472.CAN-15-2870 PMC5621135

[57] Wang X, et al. Cloning and variation of ground state intestinal stem cells. Nature. 2015;522(7555):173–8. 26040716 10.1038/nature14484 PMC4853906

[58] Meran L, Baulies A, Li VSW. Intestinal stem cell niche: the extracellular matrix and cellular components. Stem Cells Int. 2017;2017:7970385. 28835755 10.1155/2017/7970385 PMC5556610

[59] Onfroy-Roy L, et al. Extracellular matrix mechanical properties and regulation of the intestinal stem cells: when mechanics control fate. Cells. 2020;9(12). 10.3390/cells9122629 PMC7762382 33297478

[60] Gu W, et al. SATB2 preserves colon stem cell identity and mediates ileum-colon conversion via enhancer remodeling. Cell Stem Cell. 2022;29(1):101-115.e10. 10.1016/j.stem.2021.09.004. Epub 2021 Sep 27. 10.1016/j.stem.2021.09.004 PMC8741647 34582804

[61] Girish N, et al. Persistence of Lgr5+ colonic epithelial stem cells in mouse models of inflammatory bowel disease. Am J Physiol Gastrointest Liver Physiol. 2021;321(3):G308–24. 34260310 10.1152/ajpgi.00248.2020 PMC8461791

[62] Parikh K, et al. Colonic epithelial cell diversity in health and inflammatory bowel disease. Nature. 2019;567:49–55. 30814735 10.1038/s41586-019-0992-y

[63] Markandey M. et al. Gut microbiota: sculptors of the intestinal stem cell niche in health and inflammatory bowel disease. Gut Microbes. 2021;13. 10.1080/19490976.2021.1990827 PMC8583176 34747326

